# Blood Biomarkers of Neonatal Sepsis with Special Emphasis on the Monocyte Distribution Width Value as an Early Sepsis Index

**DOI:** 10.3390/medicina59081425

**Published:** 2023-08-04

**Authors:** Murad A. Mubaraki, Ayman Faqihi, Fatmah AlQhtani, Taghreed A. Hafiz, Ahmed Alalhareth, Felwa A. Thagfan, Sherif Elshanat, Rewaida A. Abdel-Gaber, Mohamed A. Dkhil

**Affiliations:** 1 Clinical Laboratory Sciences Department, College of Applied Medical Sciences, King Saud University, Riyadh 12372, Saudi Arabia; mmubaraki@ksu.edu.sa (M.A.M.);; 2 Pathology Department, King Saud University Medical City (KSUMC), Riyadh 12372, Saudi Arabia; 3 Ministry of Health, Najran 44561, Saudi Arabia; 4 Department of Biology, College of Science, Princess Nourah Bint Abdulrahman University, Riyadh 11671, Saudi Arabia; 5 Department of Parasitology, Faculty of Veterinary Medicine, Alexandria University, Alexandria 22758, Egypt; 6 Department of Zoology, Faculty of Science, Cairo University, Giza 12613, Egypt; rewaida@sci.cu.edu.eg; 7 Department of Zoology and Entomology, Faculty of Sciences, Helwan University, Cairo 11795, Egypt; 8 Applied Science Research Center, Applied Science Private University, Amman 11931, Jordan

**Keywords:** neonatal sepsis, MDW, haematological parameters

## Abstract

*Background and Objectives*: Early detection of neonatal sepsis is critical because it is potentially fatal. Therefore, sepsis biomarkers of sufficient sensitivity and specificity are needed. This study aimed to evaluate the utility of peripheral blood parameters as neonatal sepsis biomarkers and the diagnostic performance of the monocyte distribution width (MDW) in sepsis in a neonatal intensive care unit. *Materials and Methods*: A cross-sectional study was conducted from September 2019 to August 2020 at the King Saud University Medical City in Riyadh, Saudi Arabia. Samples were collected and organised as follows: 77 study cases were subdivided into two subgroups (other health complication (49) and sepsis (28)), and there were 70 controls. The causative microorganisms of neonatal sepsis were isolated. Peripheral blood samples were collected from each neonate in an ethylenediaminetetraacetic acid tube for a complete blood count and a leukocyte differential count. Moreover, the receiver operating characteristic (ROC) curve analysis was used to measure the diagnostic performance of the MDW. *Results*: The haematological parameters and neonatal sepsis cases had a considerable correlation. The MDW was the most significant haematological parameter. The ROC analysis of the MDW demonstrated that the area under the curve was 0.89 (95% confidence interval: 0.867 to 0.998) with a sensitivity of 89.3%, a specificity of 88.2%, and a negative predictive value of 97.2% at the cut-off point of 23. *Conclusions*: The use of haematological parameters is feasible and can be performed rapidly. Neonatal sepsis showed a strong correlation with leukopenia, anaemia, thrombocytopenia, and an elevated MDW value. Moreover, the ROC curve analysis confirmed the high diagnostic ability of the MDW in neonatal sepsis prediction.

## 1. Introduction

Neonatal sepsis is a common cause of morbidity and mortality among newborns [1]. It presents nonspecific clinical signs and symptoms, such as respiratory distress, hypotension, apnoea, patent duct arteriosus, and necrotizing enterocolitis; thus, it is difficult to diagnose [2,3]. Sepsis is caused by the extreme response of the host to infection. Furthermore, neonatal sepsis has been defined as an infection of a sterile site (e.g., blood, urine, or cerebrospinal fluid) accompanied by a systemic inflammatory response that can manifest within the first 72 h of life (early-onset sepsis). Symptom onset in late-onset sepsis occurs at >72 h of life [4].

One study performed in Saudi Arabia demonstrated that the main cause of neonatal sepsis in prematurity and caesarean operation cases was Gram-negative bacteria, which was also the main cause of deaths in early-onset neonatal sepsis (EONS) and late-onset neonatal sepsis (LONS). Moreover, leucocytosis, higher C-reactive protein (CRP), and thrombocytopenia were reportedly significant sepsis markers, especially in LONS [5]. Meanwhile, two studies investigated the colonization rate of group B streptococcus in pregnant women and women in labour [6,7]. Several types of pathogens are incriminated in pathogenic sepsis. Although bacterial infection is still the main cause of pathogenic sepsis, viral and fungal infections represent an important percentage of sepsis aetiologies, especially in immunocompromised patients [8,9]. The World Health Organisation classified sepsis as a global health priority in 2021 [10]. Moreover, each hour of delay in treatment raises the mortality burden of sepsis between 7% and 10% [11]. Thus, early and rapid diagnosis of sepsis is crucial. However, the diagnosis of patients at risk of sepsis is challenging because it is a dynamic disease with multiple inflammatory responses based on different pathogens and immune statuses [12]. Several studies have been conducted to detect reliable biomarkers for predicting outcomes and evaluating treatment responses; however, not all are available for routine clinical use [13]. Nevertheless, the most common biomarkers employed in sepsis diagnosis are the CRP and procalcitonin (PCT) [3].

In addition to the traditional complete blood count (CBC) parameters, a new haematological parameter analyser has emerged, which is called cell population data (CPD). The monocyte distribution width (MDW) is a CPD parameter that reflects the morphological and functional characteristics of monocytes (MO) [14]. Moreover, the Food and Drug Administration recently approved the MDW as an early sepsis indicator [15]. Since MOs are one of the first responders against infection, the MDW was proposed to be a novel biomarker of sepsis in the ED under the definition of Sepsis 2 criteria [15]. Several studies have been conducted to prove the diagnostic performance of the MDW in predicting sepsis, especially in the ED and intensive care unit [16,17,18], and they have shown good results. Furthermore, the diagnostic performance of the MDW, CRP, and PCT as predictors of sepsis have been studied in association and individually, and the results have shown that the diagnostic ability of the MDW was not lower than those of the CRP and PCT in terms of the areas under the curve (AUCs) [16]. However, the CRP is insensitive and non-specific for LONS diagnosis [19]. By contrast, the PCT’s discriminative value plays a role in detecting the severity and mortality of LONS. This study aimed to identify more sensitive and applicable peripheral haematological biomarkers in neonatal infection.

## 2. Materials and Methods

### 2.1. Study Design, Setting, and Population

A cross-sectional study was conducted at the King Saud University Medical City (KSUMC) hospital neonatal intensive care unit (NICU) in Riyadh, Saudi Arabia from September 2019 to August 2020. The study included 147 Saudi Arabian neonates (72 males and 75 females) with a mean age of 3.17 days at the admission time to NICU. Neonatal blood samples were categorized into the following: the control group, which included neonates with normal blood values and who were free from health complications, and the study group, which included neonates who were admitted with signs of sepsis and abnormal blood values. In the study group, the samples were subdivided according to their susceptibility to developing sepsis: the sepsis group and the other health complication (OHC) group.

The OHC is defined in the current study as comprising cases with negative results for blood culture and other developed health problems, such as respiratory distress syndrome, jaundice, decreased activity, low birth weight, transient tachypnoea of the newborn, hyperglycaemia, and hypoglycaemia. At study initiation, every taken blood sample was first subjected to a blood culture assay to be confirmed as whether it is sepsis, along with suggestive clinical signs. Septic microorganisms were isolated from blood culture assay using the BD Bactec analyser (blood culture system). Among the 147 newborns, 77 blood samples from neonates (39 males and 38 females) born at KSUMC had clinical symptoms and sepsis as determined by a blood culture assay; they were therefore included in the study group. In the control group, a total of 70 blood samples from healthy newborns (36 males and 34 females) born at KSUMC were collected (Table 1).

### 2.2. Biomarkers

Two-millilitre samples of venous blood from each neonate from each group were collected in ethylenediaminetetraacetic acid microtainer tubes for CBC assay and leucocyte differential count (white blood cell count (WBC), red blood cell count (RBCs), haemoglobin (Hgb), haematocrit (Hct), mean cell volume (MCV), mean corpuscular haemoglobin (MCH), red cell distribution width (RDW), platelet (PLT), mean platelet volume (MPV), MDW, neutrophil (NE), lymphocyte (LY), eosinophil (EO), MO, and basophil (BA)). Not Applicable (N/A), CBC and leucocyte differential count were performed and analysed on a full automated haematological analyser (Unicel DxH800 analyzer) (Beckman coulter Inc., Miami, FL, USA) Version 3.0 software (Beckman Coulter 2009, Miami, FL, USA).

### 2.3. Inclusion and Exclusion Criteria

All the inclusion and exclusion markers are summarized in Table 2.

### 2.4. Statistical Analyses

Statistical analysis was performed using IBM SPSS Statistics for Windows, version 22 (IBM Corp., Armonk, NY, USA) and GraphPad Prism version 8.0 (GraphPad, Boston, MA, USA) for windows. Continuous variables are reported as mean ± standard error of the mean (SEM) according to their group. Categorical variables with corresponding percentages were identified as frequencies. An unpaired *t*-test (independent sample *t*-test) was used to detect differences in the categorical variables between the groups. The diagnostic ability of a haematological parameter was assessed based on sensitivity, specificity, predictive positive value (PPV), and predictive negative value (PNV), which were computed as follows:

(1) $$sensitivity=\frac{true\;positive}{true\;positive+false\;negative},$$

(2) $$specificity=\frac{true\;negative}{true\;negative+false\;positive},$$

(3) $$PPV=\frac{true\;positive}{true\;positive+false\;positive},$$

(4) $$PNV=\frac{true\;negative}{true\;negative+false\;negative}.$$

Moreover, the chi-square test depicting the correlation of sepsis with the haematological aspects was performed in this study. Additionally, the AUC for haematological parameters and established cut point for MDW was determined through receiver operating characteristic (ROC) curve analysis. A *p*-value of <0.05 was considered significant for all statistical parameters.

## 3. Results

### 3.1. Patient Characteristics

A total of 147 Saudi Arabian neonates admitted at the NICU at KSUMC were enrolled in this study. A total of 70 neonates were enrolled into the control group, while 77 neonates were included in the study group, which was later subdivided into the sepsis (28) and OHC (49) groups (Table 1). Among the samples, 72 (48.98%) were from males, and 75 (51.02%) were from females. The neonate average age was 3.17 days. Among the 147 neonates, 63 were full-term (42.86%), and 84 were pre-term (57.14%). The average gestation age was ≈36 weeks.

Based on the blood culture assay and the examination of the suggestive clinical signs, 70 (47.6%) neonates fell within the control group (Table 1). On the contrary, the study group included 77 (52.4%) individuals. Furthermore, within the study group, 28 developed sepsis, which represents 19% of the total studied sample size, whereas 49 developed other health complications, representing 33% of the total studied sample size. Interestingly, the mean age of the sepsis group was 8.5 days with an equal sex distribution, i.e., 50% male and 50% female (Table 1).

### 3.2. Pathogens and Blood Culture

In the conducted study, blood samples were collected from the neonates and analysed for the presence of pathogens through blood culture tests. Analysis of the data showed that 28 (19.0%) of the total samples and 36.37% of those in the study group were positive for sepsis; 4 of these neonates were confirmed based on clinical manifestations (Table 1 and Table 3). Gram-positive organisms were more commonly seen in the sepsis group. The most common isolated pathogens were *Staphylococcus* spp., *E. coli*, *Streptococcus* spp., *Klebsiella pneumoniae*, *Enterobacter cloacae*, and *Acinetobacter baumannii*. Furthermore, 15 (53.6%) out of the 28 sepsis cases were EONS (early onset ≤ 72 h) and 13 (46.4%) were LONS (late onset > 72 h) (Table 3).

### 3.3. Relationship between Peripheral Blood Biomarkers and Neonatal Infections

Generally, analysing the CBC and leucocyte differential count in each group (control and study group) showed that most of the haematological parameters were significantly altered except for MCV, MCH, RDW, PLT, and MPV. The mean ± SEM values of the parameters (WBC, RBC, Hgb, and Hct) of the study group were 12.16 ± 0.91, 4.23 ± 0.10, 150.80 ± 3.71, and 44.93 ± 1.10, respectively, which showed a significant reduction compared to that of the control group, which have mean ± SEM values of 15.98 ± 0.70, 4.88 ± 0.08, 176.00 ± 2.38, and 52.10 ± 0.71, respectively (Table 4). Regarding the differential leucocyte count, data analysis results showed a considerable increase in LY% and MDW values (35.58 ± 2.42 and 24.37 ± 0.82, respectively) in the study group than in those in the control group (27.84 ± 1.72 and 18.73 ± 0.22, respectively). By contrast, NE% significantly decreased (49.18 ± 2.51) in the study group compared with that in the control group (59.38 ± 1.79) (Table 4).

Statistical comparison of both groups (sepsis and OHC groups) showed that no essential differences were reported in both groups in some biomarkers, such as WBCs, PLT, MCHC, NE, MO, EO, BA, and the NE-LY ratio. By contrast, several other haematological biomarkers showed considerable significance, especially MDW. The data analysis showed that MDW was highly significant in the sepsis group (average of MDW = 31.04) compared to that in the OHC group (average of MDW = 20.56). Moreover, a significant reduction was shown in several haematological parameters in the sepsis group compared to that in the OHC group, such as RBC (3.96 in the sepsis and 4.39 in the OHC groups), Hgb (134.6 in the sepsis and 160.14 in the OHC groups), Hct (40.22 in the sepsis and 47.62 in the OHC groups), MCV (101.47 in sepsis and 109.02 in OHC group), and MCH (33.8 in the sepsis and 36.93 in the OHC groups). Conversely, a significant increase was shown in MPV values and MDW, since the MPV value was 8.85 in the sepsis group, higher compared with that in the OHC group (7.78) (Table 5).

### 3.4. Diagnostic Performance of WBC Count, Differential WBC, MDW, and Haematological Parameters in Predicting Sepsis

To elucidate the diagnostic role of biomarkers as early potential predictors of neonatal sepsis, the haematological parameters of the sepsis group were compared against those of the control and OHC groups.

#### 3.4.1. WBC and Leucocyte Differential Count Biomarkers

Data analysis results showed that the leucocyte count of 57.1% of the neonates from the sepsis group was significantly impacted. On the contrary, 97.1% and 61.2% of the neonates from the control and OHC groups, respectively, recorded a normal range of the number of leucocytes (9–25 × 10^3^/µL). Therefore, because of the significant correlation between WBC count and sepsis, WBC count is proposed to be a predictor of the early diagnosis of sepsis (Table 6). In the same context, NE% had a considerable alteration in neonates with sepsis, in which 46.1% of neonates from the sepsis group had either neutrophilia (17.9%) or leukopenia (28.6%). Moreover, a high MDV value (>23) was recorded in 89.3% of the neonates with sepsis. Meanwhile, 98.6% and 73.5% of the neonates from the control and OHC groups, respectively, were found to have low MDW (<23). Thus, the MDV value may be used as a potential biomarker in predicting neonatal sepsis.

#### 3.4.2. RBC Indices

Concerning RBC and its relating parameters, 50% of neonates from the sepsis group displayed a significantly reduced RBC count. Meanwhile, a normal RBC count was recorded in 95.7% of neonates in the control group and 77.6% of neonates in the OHC group. Hence, the RBC count may be used as an early sepsis biomarker.

The concentration of Hgb in 57.1% of neonates from the sepsis group significantly decreased to <137 g/L, while it was within the normal range in 94.3% of neonates from the control group and 81.6% of neonates from the OHC group. Thus, Hgb may also be a potential ESI.

The comparison of the HCT value of individuals in the sepsis group against that of those in the control and OHC groups showed that the HCT value significantly correlated to the diagnosis of neonatal sepsis. Moreover, 67.9% of neonates from the sepsis group displayed a low percentage of HCT in contrast to that of those in the control and OHC groups that showed normal HCT values. Hence, HCT may be an additional index of neonatal sepsis (Table 7).

#### 3.4.3. Platelet Biomarkers

Meanwhile, 46.4% of the neonates from the sepsis group revealed thrombocytopenia (PLT < 175), while 87.1% of the neonates from the control group and 79.6% of the neonates from the OHC group displayed a normal PLT range (175–326 × 103/µL). Statistical analysis found a positive significant correlation between PLT and sepsis. Thus, PLT may be a sepsis diagnostic biomarker. However, a negative correlation between sepsis and MPV was observed (Table 8).

### 3.5. Comparison between the Predictive Values of MDW and Other Biomarkers

**Figure 1:**
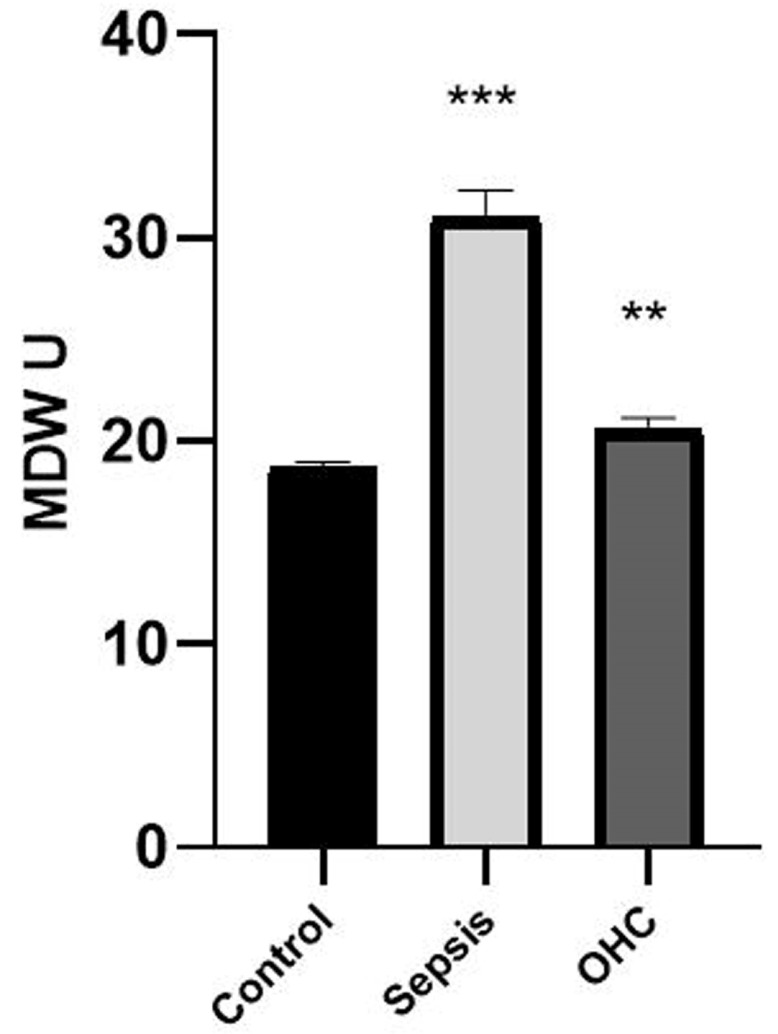
The monocyte distribution width value in the neonate samples was significantly increased in the study group, mainly in the sepsis and other health complication groups compared to that in the control group. Bars represent the mean ± standard error of the mean. *** represents statistical significance at *p* < 0.001 and ** indicates statistical significance at *p* < 0.01 (independent sample *t*-test).

MDW reflected a diagnostic ability comparable to those of another traditional haematological biomarker (WBC, RBC, PLT). Meanwhile, 89.3% of the neonates from the sepsis group presented an MDW value over 23. Consequently, MDW might be valid as an alternative biomarker (Figure 1).

#### 3.5.1. ROC Curve Analysis for Sepsis Prediction

**Figure 2:**
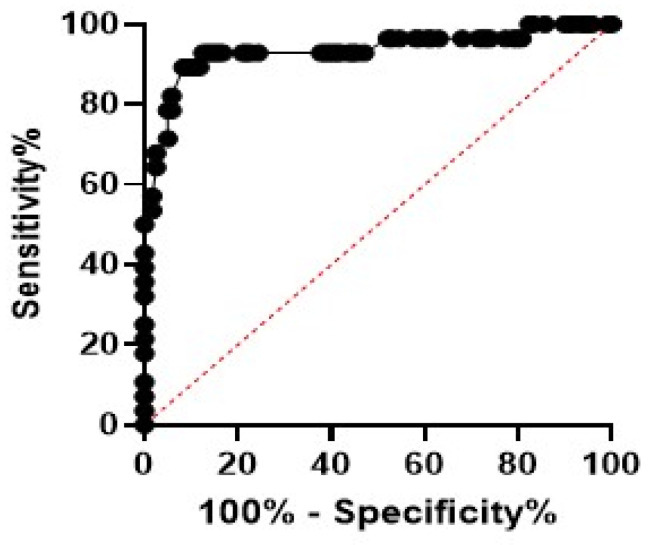
Receiver operating characteristic (ROC) curve showed that the monocyte distribution width (MDW) was significant in neonatal sepsis. The best estimated MDW was 23 as depicted by the ROC curve. Sensitivity was 89.3%, and specificity was 88.2%.

According to the data analysis, MDW was found to be superior to other haematological parameters regarding sepsis. To measure the diagnostic accuracy of MDW as a potential biomarker for the prediction of sepsis, ROC curve analysis (AUC) was performed, and results showed the high diagnostic performance of MDW in AUC (0.89, 95% confidence interval: 0.867 to 0.998) (Table 9), a cut-off value of 23, the highest sensitivity (89.3%), specificity (88.2%), and negative predictive value (97.2%) (Figure 2).

#### 3.5.2. Impact of Antibiotic Therapy in Restoring the MDV Value

**Figure 3:**
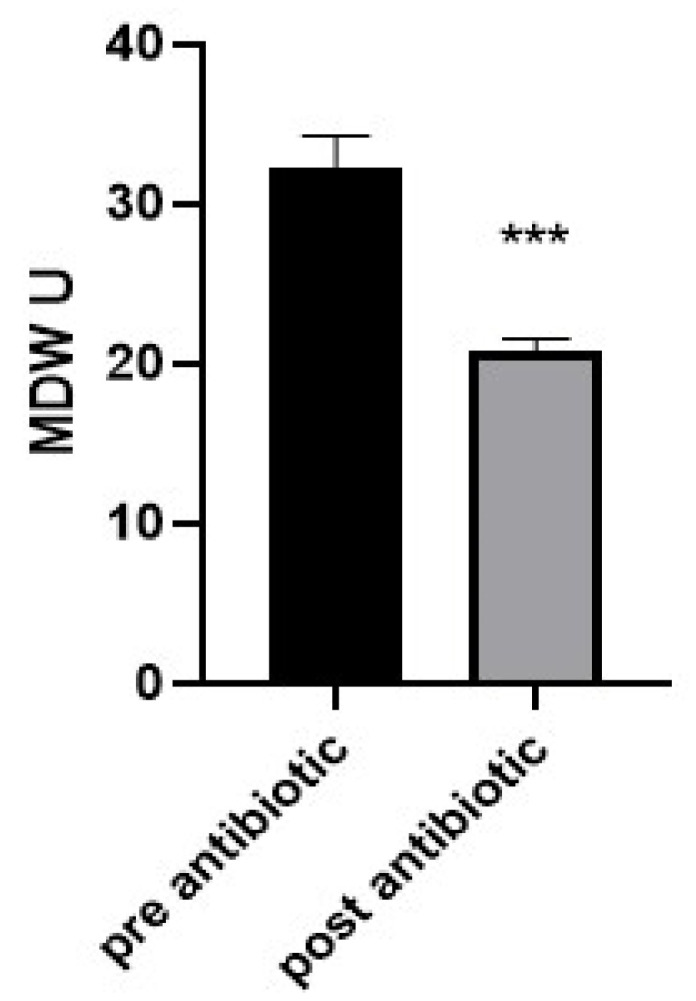
Analysis of the neonate samples that had sepsis shows that monocyte distribution width significantly declined in the post-antibiotic group compared to that in the pre-antibiotic group. Bars represent the mean ± standard error of the mean. *** indicates statistical significance at *p* < 0.001 (independent sample *t*-test).

To evaluate the changes in the MDW value after antibiotic intervention in neonates with sepsis, a comparison was carried out on 10 neonates (6 male and 4 female) with sepsis before and after antibiotic treatment. The results showed that a significant reduction in MDV value was recorded after the treatment. The mean MDW in the pre-antibiotic treatment was 32.39 and 20.74 in the post-antibiotic treatment (Figure 3).

## 4. Discussion

Sepsis is a life-threatening disease with widely variable symptoms, ranging from silent signs during the early stages [19] to severe and sudden [20]. In this study, baseline characteristics that may shape the risk factors of neonatal sepsis occurrence [21] were considered.

Bacterial infection remains a substantial cause of neonatal sepsis. In the current study, Gram-positive organisms (62.5%) were the predominant pathogens, while Gram-negative organisms (37.5%) were less frequent. This is consistent with a study that included eight Arabic countries and reported that Gram-positive organisms were the primary cause in Saudi Arabia, Bahrain and Kuwait, and the United Arab of Emirates. Contrarily, a Gram-negative organism was the main cause in Egypt, Iraq, Jordan, and Libya [22]. Therefore, we aimed to identify the more sensitive and applicable peripheral haematological biomarkers in the neonatal infection.

Blood culture has long been the gold standard, although it is time consuming in the diagnosis of sepsis. Moreover, many clinicians often start antibiotic treatment, although the culture results are undecided. Additionally, acute reactant protein (CRP or PCT) is usually a useful marker for septic disease diagnosis, but its short half-life is a drawback. Therefore, many studies have been performed to determine reliable sepsis biomarkers.

Hundreds of biomarkers for predicting sepsis are currently being discussed [23]. However, many limitations remain, such as sensitivity, specificity, diagnostic performance, elevated cost, and time required. Moreover, these biomarkers may have less significant results due to some inflammatory conditions in the absence of sepsis [18,24,25].

Generally, our results showed that most of the haematological parameters (RBC count, Hgb, HCT, MCV, MCH, PLT count, WBC count, MDW, NE%) were significantly correlated with sepsis. However, our results emphasised the greatness of MDW as a pre-diagnostic tool like a haematological parameter. This was similar to other findings [15,26]. Nonetheless, this finding supports the use of MDW as a screening haematological parameter [18]. Combining WBC count with MDW was an effective predictor of sepsis. This confirms the idea that there is no one parameter that combines the required sensitivity and specificity to precisely diagnose sepsis [27,28]. Furthermore, almost half of the confirmed sepsis cases had decreased WBC counts (leukopenia). Christoph et al. [29] demonstrated a statistically significant association between WBC count and sepsis. In the same context, leukopenia is more indicative than leucocytosis; however, blood samples should be taken within 4 to 6 h of stimulation because the number of WBCs increases in the late stage of sepsis, which is one of the drawbacks of WBCs [30,31,32].

Overall, the cut-off MDW value was 23, lower than the estimated value (26.63). The MDW value might vary according to the clinical department where the study was conducted, as well as the degree of severity of the cases [33]. Severe thrombocytopenia was detected in the dominant sepsis cases, whereas moderate and mild thrombocytopenia occurred in the lowest and average sepsis cases, respectively. Thus, PLT may be a decent marker in sepsis, which is consistent with other findings [34].

Furthermore, reduced RBC count, Hgb concentration, MCV, and MCH values were clearly significant in more than half of the sepsis cases, in agreement with previous outcomes [35]. In the same context, CBC is considered an acceptable tool in case of sepsis as it is routinely ordered as the first diagnostic test in any clinic department. Moreover, it is feasible, not expensive, and requires less time [18]. By contrast, CBCs were considered poor diagnostic markers because of their poor sensitivity [29].

The noteworthy drop in MDW values after the antibiotic treatment is significant. MDW represents a significant association between disease severity, mortality, and treatment outcome among sepsis survivors in comparison with non-survivors, highlighting the association between MDW and mortality [33]. Despite its modest sensitivity and low false-positive rate, this study confirmed that MDW dramatically improves diagnostic performance by enhancing its specificity without compromising its sensitivity [18].

The key peripheral blood parameters as newborn sepsis indicators between the healthy group, sepsis, and OHC groups are comprehensively analysed in the current study, which is its strength. However, because we only included data from one institution, our sample size was limited. To more thoroughly analyse the variations between early-onset and late-onset sepsis, a larger sample size would be helpful.

## 5. Conclusions

Numerous haematological markers including MDW are used to assess the majority of adult sepsis cases. However, a variety of haematological measures other than MDW are used to assess the majority of newborn sepsis cases. Therefore, this study suggested MDW as a biomarker for early neonatal sepsis. Because CBC and leukocyte differential count tests are practical and quick, more people are using haematological parameters, which leads to earlier treatment initiation and lower mortality. However, subsequent research including a sizable patient sample must thoroughly examine the function of MDV in newborn sepsis.

## Figures and Tables

**Table 1:** Baseline characteristics of the neonates for control and case groups.

Characteristic	Description	Control = 70	Study Group *n* = 77		Study Cases *n* = 77
			Other Health Complications *n* = 49	Sepsis *n* = 28	
Blood Culture	Negative	70 (100.0%)	49 (100.0%)	4 (14.29%)	53 (68.83%)
	Positive	0 (0.0)	0 (0.0)	24 (85.71%)	24 (31.17%)
Gender	Female	34 (48.57%)	24 (49%)	14 (50%)	38 (49.35%)
	Male	36 (51.43%)	25 (51%)	14 (50%)	39 (50.65%)
Preterm infant	no	41 (58.57%)	17 (34.69%)	5 (17.86%)	22 (28.57%)
	yes	29 (41.43%)	32 (65.31%)	23 (82.14%)	55 (71.43%)
Gestation age (week)	Mean ± SD	37.17 ± 3.34	35.18 ± 4.22	32.86 ± 4.55	34.34 ± 4.55
	min–max	30–41	24–40	24–41	24–41
Age (day)	Mean ± SD	2.04 ± 2.67	2.70 ± 5.50	8.51 ± 7.21	4.17 ± 6.15
	min–max	1–12	1–26	1–24	1–26
Baby is well	N/A	70 (100%)	0 (0.0)	0 (0.0)	0 (0.0)

**Table 2:** Inclusion and exclusion criteria.

Criteria	Inclusion	Exclusion
Nationality of neonates	Saudi	Non-Saudi
Specimen	Probable specimen	Clotted specimen
Anticoagulant tube	EDTA tube for haematological	Other tubes
Age	<28 days	≥28 days
Order	CBC and diff	CBC only
Antibiotics	Not started antibiotics	Started antibiotics

**Table 3:** The types of isolated pathogens and sepsis.

Characteristics	Description	Control *n* = 70	Case *n* = 77
Sepsis	No	70 (100.0%)	49 (63.63%)
	Yes	0 (0.0)	28 (36.37%)
Sepsis type	Early onset ≤ 72 h	0 (0.0)	15 (53.6%)
	Late onset > 72 h	0 (0.0)	13 (46.4%)
Organisms	Gram-negative	0 (0.0)	9 (37.5%)
	Gram-positive	0 (0.0)	15 (62.5%)
Isolation of the pathogens	*Escherichia coli*	0 (0.0)	5 (6.50%)
	*Enterobacter cloacae*	0 (0.0)	1 (1.30%)
	*Klebsiella pneumoniae*	0 (0.0)	2 (2.6%)
	Methicillin-resistant *Staphylococcus aureus* (MRSA)	0 (0.0)	1 (1.30%)
	*Staphylococcus aureus*	0 (0.0)	4 (5.19%)
	*Staphylococcus epidermidis*	0 (0.0)	6 (7.79%)
	*Streptococcus agalactiae*	0 (0.0)	4 (5.19%)
	*Acinetobacter baumannii*	0 (0.0)	1 (1.30%)

**Table 4:** Peripheral blood biomarker differences between study and control groups.

Characteristic	Description	Control (*n* = 70)	Study (*n* = 77)	*p* Value
White blood cells (×103/µL)	Mean ± SEM	15.98 ± 0.70	12.16 ± 0.91	
	min–max	9.0–30.0	1.5–31.90	<0.001
Red blood cells (×106/µL)	Mean ± SEM	4.88 ± 0.08	4.23 ± 0.10	
	min–max	3.80–7.40	2.50–6.0	<0.001
Haemoglobin (g/L)	Mean ± SEM	176.00 ± 2.38	150.80 ± 3.71	
	min–max	135.0–234.0	73.0–207.0	<0.001
Haematocrit (%)	Mean ± SEM	52.10 ± 0.71	44.93 ± 1.10	
	min–max	40.10–67.80	21.30–60.10	<0.001
Red cell distribution width (%)	Mean ± SEM	16.97 ± 0.13	17.76 ± 0.32	
	min–max	14.60–20.4	14.0–33.40	0.03
Neutrophil %	Mean ± SEM	59.38 ± 1.79	49.18 ± 2.51	0.001
	min–max	13.0–86.10	3.70–90.0	
Lymphocytes %	Mean ± SEM	27.84 ± 1.72	35.58 ± 2.42	0.01
	min–max	9.80–84.0	3.10–84.90	
Monocyte Distribution Width	Mean ± SEM	18.73 ± 0.22	24.37 ± 0.82	<0.001
	min–max	14.90–24	15.0–42.0	

**Table 5:** Comparison between other health complication (OHC) group and sepsis group on haematological parameters.

Characteristic	Other Health Complication (*n* = 49)		Sepsis (*n* = 28)		*p* Value
	Min–Max	Mean ± SEM	Min–Max	Mean ± SEM	
Red blood cells (×106/µL)	2.6–6.0	4.39 ± 0.11	2.5–6.0	3.96 ± 0.17	0.03
Haemoglobin (g/L)	103–207	160.14 ±3.89	73–200	134.6 ± 6.66	<0.001
Haematocrit (%)	30.10–60.1	47.62 ± 1.15	21.3–59.5	40.22 ± 2.0	<0.001
Mean cell volume (fL)	95–135.9	109.02 ±1.19	83.0–127.0	101.47 ± 1.78	<0.001
Mean corpuscular haemoglobin (pg)	31.6–48.10	36.93 ± 0.43	27.0–40.3	33.88 ± 0.60	<0.001
Red cell distribution width (%)	14.0–22.6	17.42 ± 0.25	14.4–33.4	18.36 ± 0.76	0.16
Mean platelet volume (fL)	5.9–9.6	7.78 ± 0.09	5.40–11.20	8.85 ± 0.25	<0.001
Monocyte distribution width	15.0–30.0	20.56 ± 0.56	17.0–42	31.04 ± 1.30	<0.001

**Table 6:** Correlation of sepsis with WBC indices.

WBC Indices	Description	Normal Control (*n* = 70)		Other Health Complications (*n* = 49)		Sepsis (*n* = 28)		Pearson Chi Square Test		
								Value	df	*p* Value
WBC count	Normal	68	97.1%	30	61.2%	12	42.9%	42.44	4	<0.001
	Leukopenia	-	-	18	36.7%	13	46.4%			
	Leucocytosis	2	2.9%	1	2.0%	3	10.7%			
MDW	<23	69	98.6%	36	73.5%	3	10.7%	79.2	2	<0.001
	≥23	1	1.4%	13	26.5%	25	89.3%			
NE%	Normal	61	87.1%	29	59.2%	15	53.6%	25	4	<0.001
	Neutrophilia	3	4.3%	1	2.0%	5	17.9%			
	Neutropenia	6	8.6%	19	38.8%	8	28.6%			
LY%	Normal	12	17.1%	6	12.2%	5	17.9%	7.53	4	0.11
	Lymphocytosis	20	28.6%	26	53.1%	10	35.7%			
	Lymphocytopenia	38	54.3%	17	34.7%	13	46.4%			
MO%	Normal	13	18.6%	13	26.5%	7	25.0%	1.7	4	0.792
	Monocytosis	52	74.3%	32	65.3%	18	64.3%			
	Monocytopenia	5	7.1%	4	8.2%	3	10.7%			
EO%	Normal	66	94.3%	47	95.9%	26	92.9%	0.34	2	0.842
	Eosinophilia	4	5.7%	2	4.1%	2	7.1%			
BA%	Normal	51	72.9%	36	73.5%	20	71.4%	0.038	2	0.981
	Basophilia	19	27.1%	13	26.5%	8	28.6%			

**Table 7:** Correlation of sepsis with RBC indices.

RBC Indices	Description	Normal Control (*n* = 70)		Other Health Complication (*n* = 49)		Sepsis (*n* = 28)		Pearson Chi Square Test		
								Value	df	*p* Value
RBC	Normal	67	95.7%	38	77.6%	14	50.0%	39.25	4	<0.001
	High RBC count	3	4.3%	-	-	-	-			
	Low RBC count	-	-	11	22.4%	14	50.0%			
Hgb	Normal	66	94.3%	40	81.6%	12	42.9%	45.02	4	<0.001
	High Hgb conc	3	4.3%	-	-	-	-			
	Low Hgb conc	1	1.4%	9	18.4%	16	57.1%			
HCT	Normal	61	87.1%	32	65.3%	9	32.1%	37.76	4	<0.001
	High HCT	3	4.3%	-	-	-	-			
	Low HCT	6	8.6%	17	34.7%	19	67.9%			
MCV	Normal	56	80.0%	35	71.4%	14	50.0%	19.82	4	0.001
	High MCV	7	10.0%	9	18.4%	2	7.1%			
	Low MCV	7	10.0%	5	10.2%	12	42.9%			
MCH	Normal	47	67.1%	29	59.2%	21	75.0%	13.05	4	0.011
	High MCH	22	31.4%	20	40.8%	4	14.3%			
	Low MCH	1	1.4%	-	-	3	10.7%			
MCHC	Normal	70	100.0%	48	98.0%	27	96.4%	2.15	2	0.341
	High MCHC	-	-	1	2.0%	1	3.6%			
RDW	Normal	40	57.1%	25	51.0%	13	46.4%	2.35	4	0.671
	High RDW	29	41.4%	24	49.0%	15	53.6%			
	Low RDW	1	1.4%	-	-	-	-			

**Table 8:** Correlation of sepsis with PLT indices.

PLT Indices	Description	Normal Control (*n* = 70)		Other Health Complications (*n* = 49)		Sepsis (*n* = 28)		Pearson Chi Square Test		
								Value	df	*p* Value
PLT count	Normal	61	87.1%	39	79.6%	13	46.4%	20.74	4	<0.001
	thrombocytosis	2	2.9%	-	-	2	7.1%			
	thrombocytopenia	7	10.0%	10	20.4%	13	46.4%			
MPV	Normal	58	82.9%	42	85.7%	25	89.3%	5.75	4	0.219
	High MPV	-	-	-	-	1	3.6%			
	Low MPV	12	17.1%	7	14.3%	2	7.1%			

**Table 9:** Area under the curve (AUC) between monocyte distribution width (MDW) and state of the sepsis in neonates.

Test Result Variable	Area under ROC Curve	Asymptotic 95% Confidence Interval		*p* Value
		Lower Limit	Upper Limit	
MDW	0.89	0.86	0.99	<0.001

## Data Availability

Not applicable.
